# Mixed Cellular and Antibody-Mediated Rejection—A Rare yet Morbid Occurrence

**DOI:** 10.14309/crj.0000000000001518

**Published:** 2024-09-27

**Authors:** Cristina Chiodi, Harsh Tiwari, Esther Bak, Amor Royer, Loretta Jophlin, Luis Marsano

**Affiliations:** 1 Department of Internal Medicine, University of Louisville, Louisville, KY; 2 Division of Gastroenterology, Hepatology, and Nutrition, University of Louisville, Louisville, KY

**Keywords:** liver transplant, acute rejection, cellular rejection, antibody mediated rejection, mixed rejection

## Abstract

Liver transplantation remains the definitive treatment for end-stage liver disease, yet rejection of the transplanted organ poses a significant challenge to long-term graft survival. We present a case of a 47-year-old woman who underwent liver transplantation for primary sclerosing cholangitis. Following the procedure, the patient experienced a rare phenomenon of dual rejection, characterized by both acute cellular rejection and antibody-mediated rejection. Despite initial immunosuppressive therapy, the patient's condition deteriorated. Histopathological examination revealed concurrent signs of acute cellular rejection and antibody-mediated rejection, highlighting the complexity of immune response in allograft rejection. Management involved intensified immunosuppression targeting both T-cell-mediated and antibody-mediated pathways, along with plasmapheresis to remove circulating antibodies. This case highlights an atypical presentation of rejection after transplantation. Further research is warranted to elucidate the underlying mechanisms and optimal management approaches for dual rejection in liver transplantation.

## INTRODUCTION

Acute rejection is an important cause of allograft dysfunction and can significantly affect long-term graft survival, even among patients who recover.^1^ Although the use of immunosuppressive agents has reduced the overall incidence of acute rejection, approximately 10%–30% of liver transplant patients experience some form of acute rejection.^2^ There are 2 forms of acute rejection: acute cellular rejection (ACR) and antibody-mediated rejection (AMR). While ACR is relatively common, occurring in up to 25% of patients, AMR is infrequently seen, occurring in approximately 1% of patients.^3^ The incidence of both occurring together is unknown but rarely reported in current literature. We describe one such case of concomitant ACR and AMR after liver transplantation.

## CASE REPORT

This patient case is a 47-year-old woman with cirrhosis secondary to primary sclerosing cholangitis who underwent liver transplantation from hepatitis C virus (HCV)-positive deceased donor in setting of expedited organ availability. Model for End-Stage Liver Disease score was 25 at the time of listing. The patient underwent Roux-en-Y hepaticojejunostomy and umbilical hernia repair with orthotopic liver without any reported intraoperative complications. Immediate postoperative course was complicated by biliary leak with drain placement, anastomotic stricture requiring venoplasty, and enterococcus faecium biliary infection. In addition, the patient was started on treatment of HCV with sofosbuvir-velpatasvir (Epclusa), given initial HCV RNA was elevated to 3.3 million IU/mL.

**Figure 1.**
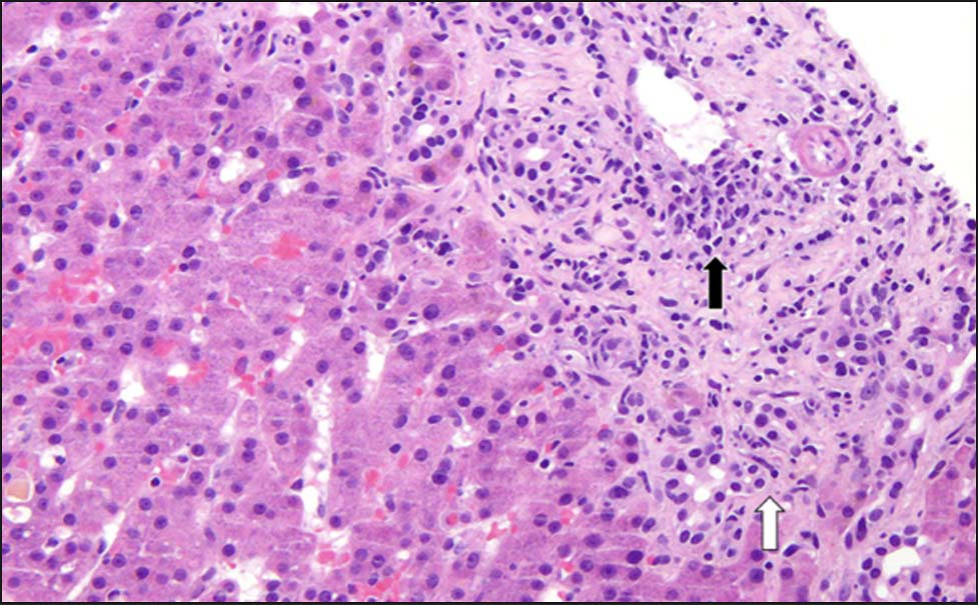
Hematoxylin and eosin (20×) section showing the portal tract. The black arrow highlights mixed portal inflammation with portal vein endotheliitis, while the white arrow shows bile ductular reaction with bile duct damage.

**Figure 2.**
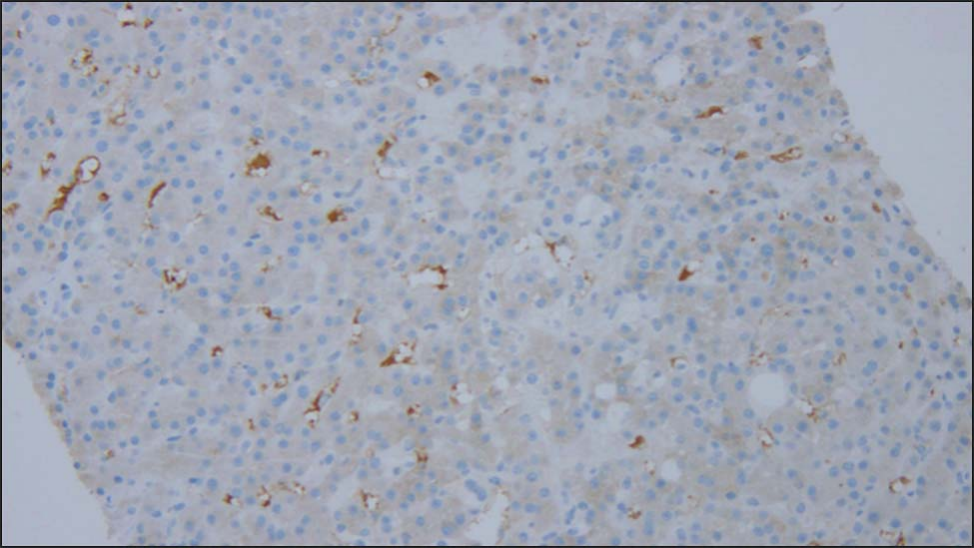
(20×) shows C4d section with evidence of linear C4d immunoreactivity in the capillaries and sinusoids.

Despite treatment of postoperative biliary complications, laboratory studies were notable for persistent cholestasis, raising concern for rejection (Table 1). Initial liver biopsy obtained on postoperative day 12 revealed active inflammation and bile duct injury consistent with ACR in addition to positive complement 4d (C4d) staining (C4d = 3) and donor-specific antibodies (DSA) concerning for AMR, which made determining a Rejection Activity Index (RAI) score difficult. Given the absence of lobular necroinflammation and lobular disarray, histologic findings were less likely to be associated with acute HCV infection (Figures 1 and 2).

**Table 1.** Depicts patient's overall hospital course, including notable events and laboratory values

Day	Event	Laboratory results				
		TB	AST	ALT	ALP	SCr
1	Orthotropic liver transplant from HCV-positive deceased donor	11.5	749	421	320	1.14
12	Transplant liver biopsy #1: portal and central inflammation consistent with acute rejection with concern for antibody-mediated component. Patient initiated on hemodialysis	27.5	197	137	127	7.06
18	Percutaneous drain placed for bile leak	27.0	997	694	193	3.48
20	Transplant liver biopsy #2: consistent with AMR and ACR	17.9	77	111	91	1.58
28	Transplant liver biopsy #3: consistent with AMR, ACR, and acute cholestasis	20.9	25	25	72	2.61
60	Transplant liver biopsy #4: ongoing ACR and AMR rejection	6.6	42	63	383	4.01
84	Discharged from hospital to inpatient rehab	2.5	20	24	258	4.10
96	Completed 3 months of HCV treatment with Epclusa Discharged from rehab to home	2.5	34	35	335	4.27
113	Readmitted to hospital for failure to thrive	2.5	34	45	451	9.25
130	Percutaneous gastric tube placed for supplemental nutrition	1.8	31	25	339	9.50
162	Transplant liver biopsy #5: no histologic evidence of rejection and nodular regenerative hyperplasia	0.8	24	13	145	3.24
176	Paracentesis with 2 liters of abdominal ascites removed. Peritoneal cultures positive for Klebsiella. Treatment initiated for intra-abdominal abscess and spontaneous bacterial peritonitis (SBP)	1.0	27	15	154	4.06
187	Transferred to ICU for undifferentiated shock	1.5	43	28	274	5.61
191	Peripheral smear showing increased schistocytes consistent with disseminated intravascular coagulation (DIC)	1.7	35	37	182	2.11
201	Esophageal biopsy showing severe ulcerations, cytomegalovirus (CMV) inclusions, and fungal yeast	5.5	60	50	276	0.54
209	Death due to septic shock with multiorgan failure	4.9	305	107	221	0.42

ACR, acute cellular rejection; ALP, Alkaline Phosphatase; ALT, Alanine Aminotransferase; AMR, antibody-mediated rejection; AST, Aspartate Aminotransferase; HCV, hepatitis C virus; ICU, intensive care unit; SCr, Serum Creatinine; TB, Total Bilirubin.

Initial treatment consisted of an immunosuppressant regimen of mycophenolate (1.5 g daily) and tacrolimus titrated to a goal tacrolimus level of 8–10 as well as continuation of oral steroids (prednisone 10 mg daily) and prophylactic coverage for opportunistic infections. The patient also completed 12 weeks of treatment with sofosbuvir-velpatasvir (400–100 mg daily), achieving a sustained virologic response. In this patient case, high-dose steroids were initially held due to severe multiorganism sepsis. Despite this regimen, subsequent biopsies continued to show evidence of multifactorial rejection. AMR treatment was escalated to alternating plasmapheresis and 10% human intravenous immunoglobulin for a total of 6 rounds of each, as well as high-dose intravenous steroids (methylprednisolone 1 g every other day) for a total of 3 doses, followed by a slow steroid taper. On postoperative day 35, treatment was escalated to 5 days of rabbit antithymocyte globulin (ATG) after successful control of bile leak. By postoperative day 42, treatment with 5 days of rituximab was started. Final biopsy on postoperative day 162 was negative for histologic evidence of active rejection, and repeat HCV viral load was negative. However, after prolonged course with significant immunosuppression, hospitalization was complicated by infection and persistent failure to thrive. The patient ultimately passed due to septic shock with multiorgan failure 7 months post-transplantation.

## DISCUSSION

ACR most commonly occurs in the early post-transplantation period and does not affect long-term graft function, if responsive to steroids. Higher risk of ACR has been associated with certain underlying liver diseases, such as primary biliary cirrhosis and hepatitis C infection. Additional risk factors that predispose patients to higher risk of cellular rejection are older age (>55 years), cytomegalovirus infection, prolonged ischemic time of allograft, and subtherapeutic levels of immunosuppression following transplantation.^4^ When ACR is suspected, biopsy is needed to confirm histopathologic changes to make the diagnosis. Histopathology of cellular rejection is typically notable for portal inflammation, bile-duct damage, and venous endothelial inflammation.^5^ Some histologic features, such as bile duct injury and portal lymphocytic infiltration, are seen in both recurrent HCV infection and ACR, therefore may need further evaluation to assess underlying etiology of graft dysfunction. Severity of histopathologic findings is graded using RAI. Mild rejection (RAI ≤ 4) is often treated by optimizing immunosuppressive agents. Moderate-to-severe rejection (RAI < 5) is treated with immunosuppression plus steroids. Severe ACR (RAI ≥5) is treated with high-dose steroids (prednisolone 200 mg or methylprednisolone 1 g for 3 days) or a high dose steroid bolus with rapid taper. These steroid regimens are effective in acute rejection in approximately 65%–80% of transplant patients.^6^ In a prior single center study analyzing factors for steroid-resistant rejection, patients with elevated bilirubin level and prior cytomegalovirus infection were most likely to be steroid nonresponders in the setting of ACR.^6^ If ACR continues to be refractory, lymphocyte depleting therapy, such as ATG, can be added to the regimen. In prior retrospective studies using ATG for steroid resistant rejection, 90% response rate was reported, however with high short-term risk for opportunistic infections and long-term risk for lymphoproliferative disorders.^7^ In addition, evaluation for other concomitant rejection etiologies and preparation of liver retransplantation should be considered.

AMR is rarely observed in liver transplantation due to the organ's innate immunity, in addition to extensive immunologic screening pretransplantation and overall improvement in immunosuppressive therapies.^8^ However, when AMR does occur, it has a predilection for patients with autoimmune liver disease.^9^ AMR is diagnosed based on histologic evidence on biopsy, positive DSA, C4d scoring, and exclusion of alternative causes. Complement activation is an important cause of tissue injury in the allograft in this form of rejection. It has been proposed that the mechanism for AMR follows a 2-hit hypothesis, where an initial insult to the allograft is made (such as ACR, viral hepatitis, hepatic ischemia), which subsequently upregulates human leukocyte antigen class II expression facilitating DSA binding, activating the classical complement cascade, and facilitating cellular cytotoxicity.^10^ Complement proteins deposited within the transplanted organ have become an important biomarker in AMR, and newer assays can measure complement activation by antibodies reactive to specific donor human leukocyte antigen expressed within the transplant.^11^ Owing to the current low level of evidence, there is not a standardized treatment of AMR in liver transplantation. However, the use of histologic features, DSA levels, and the severity of graft dysfunction can help guide a therapeutic approach. AMR is infrequently seen but when present, thought to be associated with a component of ACR. Therefore, the first-line treatment includes immunosuppressant optimization and high-dose steroids. If nonresponsive to the initial therapy, treatment can be escalated to plasmapheresis with intravenous immunoglobulin to remove circulating antibodies, then to B-cell depletion with rituximab to prevent future antibody formation.^12,13^ If all treatment options fail, retransplantation is considered.

Our patient case describes a rare occurrence of mixed rejection, ACR and AMR. While mixed rejection is not well described in current literature, this patient had multiple risk factors predisposing her to both forms of acute rejection, including an elevated risk of AMR in the setting of her history of primary sclerosing cholangitis, and predilection for ACR due to postoperative biliary complications and HCV donor infection.

## DISCLOSURES

Author contributions: C. Chiodi and H. Tiwari equally contributed to literature review and writing of this case report. H. Tiwari and E. Bak created Table 1, illustrating patient hospital course. E. Bak and A. Royer were the primary physicians involved in direct patient care. L. Jophlin and L. Marsano were the attending physicians who participated in both direct patient care and review of this case. C. Chiodi is the article guarantor.

Financial disclosure: None to report.

Previous presentation: Poster presented at American College of Physicians Chapter Meeting; September 15, 2023; Louisville, Kentucky.

Informed consent was obtained for this case report.

## References

[1] Levitsky J Goldberg D Smith AR . Acute rejection increases risk of graft failure and death in recent liver transplant recipients. Clin Gastroenterol Hepatol. 2017;15(4):584–93.e2. 27567694 10.1016/j.cgh.2016.07.035 PMC5326609

[2] Ronca V Wootton G Milani C Cain O. The immunological basis of liver allograft rejection. Front Immunol. 2020;11:2155. 32983177 10.3389/fimmu.2020.02155 PMC7492390

[3] Del Bello A Neau-Cransac M Lavayssiere L . Outcome of liver transplant patients with preformed donor-specific anti-human leukocyte antigen antibodies. Liver Transpl. 2020;26(2):256–67. 31612580 10.1002/lt.25663

[4] Dogan N Hüsing-Kabar A Schmidt HH Cicinnati VR Beckebaum S Kabar I. Acute allograft rejection in liver transplant recipients: Incidence, risk factors, treatment success, and impact on graft failure. J Int Med Res. 2018;46(9):3979–90. 29996675 10.1177/0300060518785543 PMC6136012

[5] Choudhary NS Saigal S Bansal RK Saraf N Gautam D Soin AS. Acute and chronic rejection after liver transplantation: What a clinician needs to know. J Clin Exp Hepatol. 2017;7(4):358–66. 29234201 10.1016/j.jceh.2017.10.003 PMC5715482

[6] Lee TY Choi HJ Seo CH Ahn J Hong TH You YK. Steroid-resistant rejection in liver transplant: A single-center study for risk factor and second-line treatment. Transpl Proc. 2022;54(2):443–9. 10.1016/j.transproceed.2021.10.019 35101321

[7] Schmitt TM Phillips M Sawyer RG . Anti-thymocyte globulin for the treatment of acute cellular rejection following liver transplantation. Dig Dis Sci. 2010;55(11):3224–34. 20238251 10.1007/s10620-010-1149-x

[8] Lee BT Fiel MI Schiano TD. Antibody-mediated rejection of the liver allograft: An update and a clinico-pathological perspective. J Hepatol. 2021;75(5):1203–16. 34343613 10.1016/j.jhep.2021.07.027

[9] Jiang H Guo H Yang B . Acute antibody-mediated rejection in liver transplant recipients with autoimmune liver disease: A clinical and pathologic study of 4 cases. J Pers Med. 2022;13(1):41. 36675702 10.3390/jpm13010041 PMC9865077

[10] Kim PT Demetris AJ O'Leary JG. Prevention and treatment of liver allograft antibody-mediated rejection and the role of the two-hit hypothesis. Curr Opin Organ Transpl. 2016;21(2):209–18. 10.1097/MOT.0000000000000275 26918881

[11] Stites E Le Quintrec M Thurman JM. The complement system and antibody-mediated transplant rejection. J Immunol. 2015;195(12):5525–31. 26637661 10.4049/jimmunol.1501686 PMC4672393

[12] Montgomery RA Loupy A Segev DL. Antibody-mediated rejection: New approaches in prevention and management. Am J Transpl. 2018;18(Suppl 3):3–17. 10.1111/ajt.14584 29292861

[13] Montano-Loza AJ Rodríguez-Perálvarez ML Pageaux GP Sanchez-Fueyo A Feng S. Liver transplantation immunology: Immunosuppression, rejection, and immunomodulation. J Hepatol. 2023;78(6):1199–215. 37208106 10.1016/j.jhep.2023.01.030

